# Supplementary material for: Prevalence and correlates of Human Papillomavirus infection in females from Southern Province, Zambia: A cross-sectional study
Source: PLoS One. 2024 Aug 1;19(8):e0299963. doi: 10.1371/journal.pone.0299963 (PMC11293658; doi:10.1371/journal.pone.0299963)
Supplement: S1 File — (PDF) [file pone.0299963.s001.pdf]

Mulungushi University

School of Medicine and Health Sciences

Ethics Review Committee

IORG*0010344* ***IRB****: 00012281* ***FWA****: 0002888* ***Email****:* ***somhsethics@mu.ac.zm***

Ref. No.: SMHS-MU1-2023-37

15th March 2023

Lweendo Muchaili

Mulungushi University

Livingstone Campus.

Dear Lweendo.

<u>RE: ETHICAL CLEARANCE OF THE STUDY PROTOCOL</u>

Reference is made to your protocol entitled, "*Prevalence and associates of Human Papillomavirus infection in females in Southern Province, Zambia*" that was submitted on 22nd February 2023.

On behalf of the Research Ethics Committee (REC) Chairperson, I wish to inform you that your protocol has been successfully reviewed and according to the reviewer's recommendations your protocol has been granted Ethical clearance based on the following conditions:

> ***Should there be need to modify or amend the approved protocol, you are required to notify the REC and submit protocol amendments for approval by quoting your REC reference number. You are further required to submit progress reports to the REC twice a year and a final report at the end of your study. You must report serious adverse events related to the conduct of the study and any unforeseen circumstances to the REC.***

You are now required to submit your protocol to National Health Research Authority (NHRA) for authorization following the link: https://www.nhra.org.zm/

This approval is valid for a period 15th March 2023 to 15th March 2024.

The Committee wishes you success in the execution of your study.

Yours sincerely,

MULUNGUSHI UNIVERSITY - SOMHS _ RESEARCH ETHICS COMMITTEE

Dr. Uthman Ademola Yusuf

AG SECRETARY – MUSOMHS_REC

Cc: Chairperson – MUSOMHS_REC
