# Supplementary material for: Prevalence and correlates of Human Papillomavirus infection in females from Southern Province, Zambia: A cross-sectional study
Source: PLoS One. 2024 Aug 1;19(8):e0299963. doi: 10.1371/journal.pone.0299963 (PMC11293658; doi:10.1371/journal.pone.0299963)
Supplement: Attachment — Submitted filename: PONE-D-24-06185_reviewed .docx [file pone.0299963.s003.docx]

PLOS ONE

Prevalence and correlates of Human Papillomavirus infection in females from Southern

Province, Zambia: A cross-sectional study

--Manuscript Draft--

| Manuscript Number: | PONE-D-24-06185 |
| --- | --- |
| Article Type: | Research Article |
| Full Title: | Prevalence and correlates of Human Papillomavirus infection in females from Southern Province, Zambia: A cross-sectional study |
| Short Title: | Prevalence and correlates of Human Papillomavirus infection in females from Southern Province, Zambia |
| Corresponding Author: | Lweendo Muchaili  Mulungushi University  Livingstone, Southern Province ZAMBIA |
| Keywords: | Cervical cancer; HIV; HPV; Women Living With HIV |
| Abstract: | Background  Human papillomavirus (HPV) infection is strongly associated with cervical cancer with almost all cases being associated with the infection. Cervical cancer is the leading cause of cancer death among women in Zambia and the fourth leading cause of cancer death in women worldwide. However, there is limited data on the burden and associated factors of HPV in sub-Saharan Africa. This study aimed to determine the prevalence and correlates of HPV infection in the Southern province of Zambia.  Methods  This was a cross-sectional study conducted at Livingstone University Teaching Hospital (LUTH) among 4,612 women from different districts of the southern province being screened for HPV infection between September 2021 and August 2022.  Demographic and clinical data were collected from an existing laboratory programmatic database. Multivariable logistic regression was used to estimate the factors associated with HPV infection. Results  The study participants had a median age of 39 years [interquartile range (IQR) 30, 47]. The prevalence of HPV infection was 35.56% (95%CI). At multivariable analysis, the factors associated with a positive HPV result were younger age (adjusted odds ratio (AOR) 0.98; 95% confidence interval (CI) 0.98-0.99; p. value 0.001), having provider collected sample (AOR 2.15; 95%CI 1.66 -2.79; p. value <0.001) and living with HIV (AOR 1.77; 95%CI 1.22 – 2.55; p. value <0.002).  Conclusion  The prevalence of HPV in women in the southern province of Zambia is high, and likely influenced by age and HIV status. Additionally, the outcome of the HPV test is affected by the sample collection method. Therefore, there is a necessity to enhance HPV and cervical cancer screening, especially among people with HIV. |
| Order of Authors: | Lweendo Muchaili, MSc |
|  | Precious Simushi, MPH |
|  | Bislom C. Mweene, MSc |
|  | Tuku Mwakyoma, MSc |
|  | Sepiso K. Masenga, PhD |
|  | Benson M. Hamooya, PhD |

| Additional Information: |  |
| --- | --- |
| Question | Response |
| Financial Disclosure  Enter a financial disclosure statement that describes the sources of funding for the work included in this submission. Review the [submission guidelines](http://journals.plos.org/plosone/s/submission-guidelines#loc-financial-disclosure-statement) for detailed requirements. View published research articles from [PLOS ONE](https://journals.plos.org/plosone/) for specific examples.  This statement is required for submission and will appear in the published article if the submission is accepted. Please make sure it is accurate. | The author(s) received no specific funding for this work. |
| Funded studies Enter a statement with the following details: - Initials of the authors who received each award - Grant numbers awarded to each author - The full name of each funder - URL of each funder website - Did the sponsors or funders play any role in the study design, data collection and analysis, decision to publish, or preparation of the manuscript? |  |
| Did you receive funding for this work? |  |
| Competing Interests  Use the instructions below to enter a competing interest statement for this submission. On behalf of all authors, disclose any [competing interests](http://journals.plos.org/plosone/s/competing-interests) that could be perceived to bias this work—acknowledging all financial support and any other relevant financial or nonfinancial competing interests.  This statement is required for submission and will appear in the published article if the submission is accepted. Please make sure it is accurate and that any funding sources listed in your Funding Information | The authors have declared that no competing interests exist. |

| later in the submission form are also declared in your Financial Disclosure statement.  View published research articles from [PLOS ONE](http://journals.plos.org/plosone/) for specific examples. |  |
| --- | --- |
| NO authors have competing interests Enter: The authors have declared that no competing interests exist. Authors with competing interests Enter competing interest details beginning with this statement: I have read the journal's policy and the authors of this manuscript have the following competing interests: [insert competing interests here] |  |
| * typeset |  |
| Ethics Statement  Enter an ethics statement for this submission. This statement is required if the study involved:   - Human participants - Human specimens or tissue - Vertebrate animals or cephalopods - Vertebrate embryos or tissues - Field research   Write "N/A" if the submission does not require an ethics statement.  General guidance is provided below. Consult the [submission guidelines](http://journals.plos.org/plosone/s/submission-guidelines#loc-guidelines-for-specific-study-types) for detailed instructions. Make sure that all information entered here is included in the Methods section of the manuscript. | Ethical approval was obtained from the Mulungushi University School of Medicine and  Health Sciences Research Ethics Committee (SMHS-MU1-2023-37, dated 15th March 2023). Additionally, clearance and authorization to conduct the research were obtained from the National Health Research Authority (NHRA00001/13/04/2023, dated 13th  April 2023). Permission to conduct the study at Livingstone University Teaching Hospital was granted by the Livingstone University Teaching Hospital management. All data collected were de-identified, accessed only by the study assistants and the principal investigator, and were strictly used for research purposes only.  No consent was obtained from participants as the data were analyzed anonymously |

Format for specific study types

Human Subject Research (involving human

participants and/or tissue)

Give the name of the institutional review

board or ethics committee that approved the

study

•

Include the approval number and/or a

statement indicating approval of this

research

•

Indicate the form of consent obtained

(

written/oral) or the reason that consent was

not obtained (e.g. the data were analyzed

anonymously)

•

Animal Research (involving vertebrate

animals, embryos or tissues)

Provide the name of the Institutional Animal

Care and Use Committee (IACUC) or other

relevant ethics board that reviewed the

study protocol, and indicate whether they

approved this research or granted a formal

waiver of ethical approval

•

Include an approval number if one was

obtained

•

If the study involved

non-human primates

,

add

additional details

about animal welfare

and steps taken to ameliorate suffering

•

If anesthesia, euthanasia, or any kind of

animal sacrifice is part of the study, include

briefly which substances and/or methods

were applied

•

Field Research

Include the following details if this study

involves the collection of plant, animal, or

other materials from a natural setting:

Field permit number

•

Name of the institution or relevant body that

granted permission

•

Data Availability

Authors are required to make all data

underlying the findings described fully

available, without restriction, and from the

time of publication. PLOS allows rare

exceptions to address legal and ethical

concerns. See the

[PLOS Data Polic](http://journals.plos.org/plosone/s/data-availability)

[y](http://journals.plos.org/plosone/s/data-availability)

and

[FA](http://journals.plos.org/plosone/s/data-availability#loc-faqs-for-data-policy)

[Q](http://journals.plos.org/plosone/s/data-availability#loc-faqs-for-data-policy)

for detailed information.

Yes - all data are fully available without restriction

| A Data Availability Statement describing where the data can be found is required at submission. Your answers to this question constitute the Data Availability Statement and will be published in the article, if accepted. |  |
| --- | --- |
| Important: Stating ‘data available on request from the author’ is not sufficient. If your data are only available upon request, select ‘No’ for the first question and explain your exceptional situation in the text box. |  |
| Do the authors confirm that all data underlying the findings described in their manuscript are fully available without restriction? |  |
| Describe where the data may be found in full sentences. If you are copying our sample text, replace any instances of XXX with the appropriate details.   - If the data are held or will be held in a public repository, include URLs, accession numbers or DOIs. If this information will only be available after acceptance, indicate this by ticking the box below. For example: All XXX files   are available from the XXX database (accession number(s) XXX, XXX.).   - If the data are all contained within the manuscript and/or Supporting Information files, enter the following: All relevant data are within the manuscript and its Supporting Information files. - If neither of these applies but you are able to provide details of access elsewhere, with or without limitations, please do so. For example:   Data cannot be shared publicly because of [XXX]. Data are available from the XXX Institutional Data Access / Ethics Committee (contact via XXX) for researchers who meet the criteria for access to confidential data.  The data underlying the results presented in the study are available from (include the name of the third party | All relevant data are within the manuscript and its Supporting Information files |

| and contact information or URL).  • This text is appropriate if the data are owned by a third party and authors do not have permission to share the data.  * typeset |  |
| --- | --- |
| Additional data availability information: |  |

Manuscript	[Click here to access/download;Manuscript;HPV article PLOS](https://www.editorialmanager.com/pone/download.aspx?id=35537450&guid=617d7a6a-f27d-4463-8084-56a88ef16b81&scheme=1)

[ONE v.2.docx](https://www.editorialmanager.com/pone/download.aspx?id=35537450&guid=617d7a6a-f27d-4463-8084-56a88ef16b81&scheme=1)

# Prevalence and correlates of Human Papillomavirus infection in females from Southern Province, Zambia: A cross-sectional study

Lweendo Muchaili^1*^, Precious Simushi^1^, Bislom C. Mweene^1^, Tuku Mwakyoma^1^, Sepiso K. Masenga^1, 2^, Benson M. Hamooya^1,3,4^

1 HAND Research Group, School of Medicine and Health Sciences, Mulungushi

University, Livingstone campus, Zambia;

2 Department of Medicine, Vanderbilt University Medical Center, Nashville, Tennessee, USA.
3 School of Public Health, University of Alabama at Birmingham, Alabama, USA
4 Centre for Infectious Disease Research in Zambia, Lusaka, Zambia

**Emails:** Lweendo Muchaili muchailiL2@gmail.com – Precious Simushi precioussimushi.ps@gmail.com – Bislom C. Mweene bischarm1st@gmail.com – Tuku Mwakyoma – mwakyomatk@gmail.com Benson M. Hamooya – benmalambo@gmail.com – Sepiso K. Masenga sepisomasenga@gmail.com or smasenga@mu.ac.zm

**Address for Correspondence:**

HAND Research Group, School of Medicine and Health Sciences, Mulungushi University, Livingstone campus, Zambia.

P. O Box 60009

Livingstone, Zambia

Tel: +260 977126209

E-mail: muchailiL2@gmail.com

## Abstract

**Background:** Human papillomavirus (HPV) infection is strongly associated with cervical cancer with almost all cases being associated with the infection. Cervical cancer is the leading cause of cancer death among women in Zambia and the fourth leading cause of cancer death in women worldwide. However, there is limited data on the burden and associated factors of HPV in sub-Saharan Africa. This study aimed to determine the prevalence and correlates of HPV infection in the Southern province of Zambia.

**Methods:** This was a cross-sectional study conducted at Livingstone University Teaching Hospital (LUTH) among 4,612 women from different districts of the southern province being screened for HPV infection between September 2021 and August 2022. Demographic and clinical data were collected from an existing laboratory programmatic database. Multivariable logistic regression was used to estimate the factors associated with HPV infection.

**Results:** The study participants had a median age of 39 years [interquartile range (IQR) 30, 47]. The prevalence of HPV infection was 35.56% (95%CI). At multivariable analysis, the factors associated with a positive HPV result were younger age (adjusted odds ratio (AOR) 0.98; 95% confidence interval (CI) 0.98-0.99; p. value 0.001), having provider collected sample (AOR 2.15; 95%CI 1.66 -2.79; p. value <0.001) and living with HIV (AOR 1.77; 95%CI 1.22 – 2.55; p. value <0.002).

**Conclusion:** The prevalence of HPV in women in the southern province of Zambia is high, and likely influenced by age and HIV status. Additionally, the outcome of the HPV test is affected by the sample collection method. Therefore, there is a necessity to enhance HPV and cervical cancer screening, especially among people with HIV. **Keywords:** Cervical cancer, HIV, HPV, Women Living With HIV

## Introduction

Human papillomavirus (HPV) infection is strongly associated with cervical cancer and remains the most common pathogen associated with cancer in females ^1^. Some studies have shown that by the age of 50, about 80% of the world’s population is exposed to HPV infection thus making HPV the most common sexually transmitted infection ^2,3^. The prevalence of cervical-vaginal HPV worldwide is 9.9% with most of the burden being in low- and middle-income countries (LMICs) ^4^. The pooled HPV prevalence in Sub-Saharan Africa (SSA) is estimated to be 32.3% in women ^5^. Sub-Saharan Africa region has one of the highest HPV burdens in the world and consequently has higher cervical cancer cases

5,6.

Cervical cancer is the fourth leading cause of cancer death in women worldwide. It is estimated that 570,000 females were diagnosed with cervical cancer and 311,000 globally died of cervical cancer in 2018 ^7^. Approximately 84% of all cervical cancers and

88% of deaths due to cervical cancer occur in LMICs ^8^. About 75,000 new cases and 50,000 new cases of cervical cancer are reported every year in SSA ^9^. According to world health organization (WHO) projections, cervical cancer deaths will reach 443,000 by the year 2030 and 90% will be from the sub-Saharan region ^10^.

Cervical cancer for more than a decade has been one of the most prevalent among other types of cancer and a leading cause of cancer death in women in Zambia affecting about 97.1 per 100,000 women ^11,12^. An estimated 3000 women in Zambia are diagnosed with cervical cancer yearly ^13^. In 2018, Zambia had the third highest cervical cancer incident rate worldwide at 66.4 per 100,000 with a total of 1,839 women dying of cervical cancer

^14^. A study done by Hayumbu et al. in 2021 at Livingstone University Teaching hospital (LUTH), found the prevalence of cervical cancer among women attending the cancer clinic to be 6% with an additional 19% of the women having precancerous cervical lesions

15.

Several studies have demonstrated that HPV infection is associated with early initiation of sexual activity, multiple sexual partners, having other sexually transmitted infections,

use of oral contraceptives and smoking ^16,17^. Low socioeconomic status, education level and living with HIV have also been shown to be strongly associated with HPV infection ^18–21^. However, there is a paucity of specific data on the burden and drivers of HPV infection in SSA. The aim of this study was to determine the prevalence and correlates of HPV infection among women from Southern province using programmatic data in order to generate hypotheses for follow-up interventional studies.

## Methods

### Study design and Setting

This was a retrospective cross-sectional study conducted at Livingstone University Teaching Hospital (LUTH) in the period 5^th^ May 2023 to 24^th^ May 2023. LUTH is the regional referral hospital for the Southern province of Zambia. Southern province is the third largest province in Zambia with a population of over 2.3 million people and covering

85,283 square kilometers. The study utilized programmatic data for the period September

2021 to August 2022, during which the LUTH laboratory was the sole facility performing HPV Polymerase Chain Reaction (PCR) testing in the province. All health facilities in the region collected and sent cervical swabs to the LUTH laboratory for HPV PCR testing.

### Eligibility criteria

We included all women who had been screened for HPV infection in the period September 2021 to August 2022. We excluded all women who had missing HPV PCR results from the study.

### Study population

We counted both the electronic entries on the Laboratory Information System (LIS) and the physical forms stored in the laboratory to ascertain the total number of women tested; about 5,100 women were tested for HPV infection using PCR in this period. We excluded all women who had missing HPV PCR results from the study, subsequently remaining with a final sample size of 4, 612 women.

### Study variables

The primary outcome variable was the HPV infection. Explanatory variables included HIV status (Living with HIV or Living without HIV)*,* sample type (Provider collected or Selfcollected), visit type and entry point [Visual Inspection with Acetic Acid (VIA) routine check-up, antiretroviral therapy (ART) clinic or other hospital sections].

Visit type

Women were categorized based on their clinic visit number. Those attending the clinic for cervical cancer screening for the first time made the 1^st^ category, those visiting the clinic the second, third and fourth time made the 2^nd^, 3^rd^ and 4^th^ categories respectively.

### Data sources/ measurement

A researcher-designed quantitative tool was used to collect data from the programmatic data given by the referring health facilities. Data on the following variables were collected; HIV status, sample type, visit type, entry point and HPV result. All study participants were examined using the Aptima® HPV kit on the Hologic Panther platform. This assay is for the qualitative detection of E6/E7 mRNA transcripts for 14 high-risk HPV types (16, 18, 31, 33, 35, 39, 45, 51, 52, 56, 58, 59, 66, and 68). All data was collected and analyzed anonymously.

### Data analysis

Collected data were entered, assorted and coded then analyzed using STATA version

15. Descriptive statistics (i.e. proportions, frequency, medians and interquartile range (IQR)) were used to understand the distribution of study variables. The Shapiro-Wilk test was used to test for the normality of the continuous variable (age). A Chi-square test was used to ascertain a relationship between two categorical variables. The logistic regression model (bivariate and multivariable) was used to determine factors associated with HPV infection. A p-value of less than 0.05 was considered to be statistically significant.

## Results

### General characteristics of participants

The study comprised 4,612 female study participants with a median (IQR) age of 39 years

(30, 47). The majority (n=2,734, 59.28%) were women living with HIV (WLWH) of which

1,960 (42.50%) were referred to the cancer clinic by the ART clinic. Most participants (n=3,421, 74.18%) were visiting the cancer clinic for the first time and had their samples collected by a healthcare provider (n=2,599, 56.35%). HPV PCR was positive for 1,640 (35.56%) of the participants, see Table 1.

**Table 1** Demographic and clinical characteristics of participants

| **Variable** | **Median (IQR)** | **Frequency** | **Percent (%)** |
| --- | --- | --- | --- |
| **Age (years)** | 39 (30, 47) |  |  |
| **Visit type**  1st |  | 3,421 | 97.83 |
| 2nd |  | 5 | 0.14 |
| 3rd |  | 66 | 1.89 |
| 4th |  | 5 | 0.14 |
| **Entry point**  VIA routine checkup |  | 991 | 32.28 |
| ART clinic |  | 1,960 | 63.84 |
| Other |  | 119 | 3.88 |
| **Sample type**  Self-collected |  | 444 | 14.59 |
| Provider collected |  | 2,599 | 85.41 |
| **Living with HIV?**  No |  | 279 | 9.26 |
| Yes |  | 2,734 | 90.74 |
| **HPV result** Negative |  | 2,734 | 64.44 |
| Positive |  | 1,640 | 35.56 |

IQR: interquartile range

## Relationship between HPV infection and clinical and demographic characteristics

Women with HPV PCR positive results were significantly younger than those without (median age 37 vs 39 years, p=0.002). HPV infection was significantly higher in women who had their samples collected by a healthcare provider compared to those who had self-collected samples (36.98% vs 21.4%, p=<0.001), and in WLWHIV compared to women living without HIV (36.06% vs 25.45%, p=<0.001), Table 2.

**Table 2 Relationship between HPV infection with clinical and demographic characteristics**

**Variable**	**Number	HPV result	p-value**

| **Age (years) ), median (IQR)** | 4,228 | 37 (29, 47) | 39 (31, 47) | **0.002^w^** |
| --- | --- | --- | --- | --- |
| **Visit type** | 3,497 |  |  | 0.309 |
| **1st** |  | 1,177 (34.41%) | 2,244 (65.59%) |  |
| **2nd** |  | 0 (0) | 5 (100%) |  |
| **3rd** |  | 19 (28.79%) | 47 (71.21%) |  |
| **4th** |  | 2(40%) | 3(60%) |  |
| **Entry point** | 3,070 |  |  | 0.093 |
| **VIA** routine check-up |  | 358 (36.13%) | 633 (63.87%) |  |
| **ART** clinic |  | 684 (34.9%) | 1,276 (65.1%) |  |
| **Other** |  | 31 (26.05%) | 88 (73.95%) |  |
| **Sample type** | 3,043 |  |  | **<0.001** |
| **Self-collected** |  | 95 (21.40%) | 349 (78.60%) |  |
| **Provider collected** |  | 961 (36.98%) | 1,638 (63.02%) |  |

**Positive, 1,640 Negative, 2,972 (35.56%) (64.44%)**

**Living with HIV**	3,013

**No**	71(25.45%)	208(74.55%)	**<0.001**

**Yes**	986 (36.06%)	1,748(63.94%)

Abbreviations: IQR: interquartile range, w: Wilcoxon rank-sum test, VIA: Visual inspection with acetic acid, ART: antiretroviral therapy, HPV: Human papillomavirus, bold p-values show variables with p<0.05

## Univariable and multivariable analysis

Univariate analysis shows that an increase in age by a year had a 1% reduction in the odds of having HPV infection (OR 0.99; 95%CI 0.98-0.99; p=0.014). Women who were referred to the cancer clinic by other hospital sections had 38% reduced odds of being infected with HPV compared to those who went for routine VIA (OR 0.62; 95%CI 0.40 - 0.95; p=0.031). Women who had provider-collected samples were 2.15 times more likely to have a positive HPV result compared to those with self-collected samples (OR 2.15; 95%CI 1.69 -2.74; p=<0.001). Women who were living with HIV had a 65% increased chance of having HPV infection compared to women without HIV (OR 1.65; 95%CI 1.24 – 2.18; p=<0.001).

At multivariable analysis, an increase in age by a year had a 2% reduction in the chance of having HPV infection (AOR 0.98; 95%CI 0.98-0.99; p=0.001). Having a providercollected sample increased the odds of having a positive HPV diagnosis by 2.15 times when compared to self-collected samples (AOR 2.15; 95%CI 1.66 -2.79; p<0.001). Participants with HIVWLWHIV had 77% increased odds of having HPV infection when compared to ones without HIV (AOR 1.77; 95%CI 1.22 – 2.55; p=0.002), see Table 3.

Table 3. Univariable and Multivariable analysis of factors associated with HPV infection

| **Variable** | **Crude analysis**  **OR** | **95%CI** | **pvalue** | **Adjusted analysis**  **OR** | **95%CI** | **p-value** |
| --- | --- | --- | --- | --- | --- | --- |
| **Age (years), median (IQR)** | 0.99 | 0.98-0.99 | **0.014** | 0.98 | 0.98 - 0.99 | **0.001** |
| **Visit type**  1st | Ref |  |  |  |  |  |
| 2nd | 1.83e-06 | 0 | 0.977 | 4.64E-07 | 0 | 0.988 |
| 3rd | 0.77 | 0.45- 1.31 | 0.342 | 0.69 | 0.38 - 1.24 | 0.218 |
| 4th | 1.27 | 0.21- 7.61 | 0.793 | 0.51 | 0.05 - 5.02 | 0.57 |
| **Entry point**  VIA routine check-up | Ref |  |  |  |  |  |
| ART clinic | 0.94 | 0.80 -1.11 | 0.510 | 0.88 | 0.72 - 1.09 | 0.27 |
| Other | 0.62 | 0.40 - 0.95 | **0.031** | 0.68 | 0.39 - 1.17 | 0.169 |
| **Sample type**  Self-collected | Ref |  |  |  |  |  |
| Provider collected | 2.15 | 1.69 - 2.74 | **<0.001** | 2.15 | 1.66 - 2.79 | **<0.001** |
| **HIV status**  Negative | Ref |  |  |  |  |  |
| Positive | 1.65 | 1.24 -2.18 | **<0.001** | 1.77 | 1.22 - 2.55 | **0.002** |

## Discussion

Our study aimed to investigate the prevalence and correlates of HPV infection among female study participants visiting cancer clinics in different health facilities in the southern province. The overall prevalence of HPV infection was high among our study participants (35.56%) and it was found to be more prevalent among women with HIV. This is consistent with a study conducted in Southern Africa by Ramogola-Masire et al. ^22^. However, our findings are lower than some previous studies conducted in Zambia. A study conducted by Ng’andwe et al. with a sample size of 70 women found an overall HPV prevalence of 65%, 80% in WLWH, and 55% in seronegative women ^23^. One reason which could explain the difference is that Ng’andwe et al. study screened for 17 high-risk

HPV (hr-HPV) strains whereas our study focused on 14. Furthermore, Ng’andwe et al. used a smaller sample size compared to our study. Another study by Sahasrabuddhe et al. among WLWHIV in Zambia found hr-HPV prevalence to be 90.3% ^24^. Like the study conducted by Ng’andwe et al, Sahasrabuddhe et al. included more (18) hr-HPV types in their study. Equally, Sahasrabuddhe et al. study had a smaller sample size (145) and only included WLWHIV. Therefore, the inclusion of non-uniform hr-HPV types in the two previous studies could in part explain the differences in the findings. The comparability of our study and the two previous studies may also have been affected by time differences and changes in disease trends especially since both were done more than 10 years ago

23–26.

We observed WLWHIV that HIV infection was significantly associated with HPV infection. These findings are consistent with previous studies in the region, emphasizing the

vulnerability of WLWHIV to HPV infection ^27,28,28^. HIV infection is thought to disrupt epithelial tight junctions which may promote both the establishment of new HPV infection and progression of existing infection in the female genital tract ^29^. While HPV infection naturally clears in most of the infected women, HPV infection commonly persists in the

WLWHIV due to impaired immune responses resulting from HIV pathology ^30^. The higher prevalence of HPV infection in this population highlights the urgent need for targeted interventions, such as HPV vaccination and consistent periodic cervical cancer screening, to reduce the burden of HPV-related diseases among WLWHIV in Zambia.

Another significant finding of our study was the association between age and HPV infection. Both univariate and multivariate analyses showed a consistent trend, indicating that increasing age was associated with a reduced likelihood of having HPV infection. This finding aligns with previous studies conducted in SSA, and globally, and underscores

the importance of age as a significant factor in HPV epidemiology ^31–34^. A study in Zambia found that young women were less likely to use condoms in sexual intercourse compared to their male counterparts ^35^. Another study in South Africa showed that older women are more likely to use a condom during sexual intercourse compared to young women ^36^. Condom use has been shown to offer protection against HPV infection, thus non-condom use during sexual intercourse exposes young women to HPV women, which may in part explain the higher prevalence of HPV in young women ^37,38^. Additionally, young women are more likely to have multiple sexual partners compared to older women, which puts them at higher risk of contracting HPV infection ^39–42^. Understanding the age-specific patterns of HPV infection can inform age-targeted preventive strategies, including vaccination programs and screening guidelines ^43,44^.

Another noteworthy finding in our study was the influence of the sample collection method on HPV infection rates. Women who had their samples collected by a healthcare provider had a significantly higher prevalence of HPV infection compared to the self-collected ones. This could be because health care providers are trained and are more likely to get an accurate sample whereas patients on the other hand may not fully understand proper sample collection. These findings are not consistent with other studies that have shown the reliability of self-collected HPV samples ^45–48^. However, on the other hand, the findings agree with a few studies that found that the Hologic Panther Aptima kit-based HPV tests had less sensitivity on self-collected samples ^49,50^. While these findings cannot be used to determine the actual reliability of self-collected HPV samples, our findings suggest that the sample collection technique may impact the detection of HPV infection in the Zambian population. These findings stress the need to study the effectiveness of new diagnostic methods in different populations and settings before rolling out the methods. Furthermore, the findings have practical implications for healthcare providers and emphasize the importance of standardized protocols and training for sample collection to ensure accurate and reliable results

## Strengths and Limitations

Our study has several strengths, including a large sample size and the inclusion of WLWH, which enhances our understanding of HPV infection in a high-risk population. To our knowledge, our study is the first study with a large sample size to investigate the prevalence of HPV in Zambia. Large sample sizes are advantageous in that they are more representative of the population when compared with studies with smaller numbers.

The majority of the participants were WLWH, indicating the importance of studying HPV infection in this particular group. It is well-established that individuals with compromised immune systems, such as WLWH, are at a higher risk of acquiring HPV infection and

developing associated complications, including cervical cancer ^1,51–53^. By including a substantial number of WLWHIV in our study, we have shed light on an important aspect of HPV epidemiology within the Zambian context.

Despite the strengths, we acknowledge that our study had several limitations. Firstly, since our research utilized data provided on laboratory request forms, the study was limited to the variables provided by the laboratory forms, thus we could not include variables that would have provided a more comprehensive understanding of the factors associated with HPV infection such as the age at first coital experience, sexual partners, smoking, alcohol consumption and other underlying conditions. Secondly, we could also not perform HPV genotyping due to restricted resources, thus we could not determine the most prevalent hr-HPV types. Thirdly, our study was conducted solely within cancer clinics in a single province, which may have restricted the generalizability of our findings to the broader population of Zambia. Finally, the cross-sectional nature of our study design precludes us from establishing causal relationships between the identified factors and HPV infection.

## Conclusion

In conclusion, our study contributes to the existing body of knowledge on HPV infection in SSA, particularly in the context of Zambia. The higher prevalence of HPV infection among WLWHIV and the association between age and infection highlight the need for targeted interventions and age-specific preventive strategies. The impact of sample collection methods on HPV detection emphasizes the importance of standardized sample collection protocols for both clients and healthcare workers. Longitudinal studies and a broader representation of the population are warranted to enhance our understanding of

HPV epidemiology and inform effective preventive measures.

## Abbreviations

HIV, human immunodeficiency virus; HPV, Human papilloma virus; IQR, interquartile range; LUTH, Livingstone University Teaching Hospital; LIS, Laboratory Information System; LMICs, low- and middle-income countries; PCR, Polymerase Chain Reaction; WLWHIV, Women living with HIV; WHO, World Health Organization.

## Ethics approval and consent to participate

Ethical approval was obtained from the Mulungushi University School of Medicine and

Health Sciences Research Ethics Committee (SMHS-MU1-2023-37, dated 15th March 2023). Permission to conduct the study at Livingstone University Teaching Hospital was granted by the Livingstone University Teaching Hospital management. All data was collected and analyzed anonymously.

## Acknowledgments

Much appreciation to the Laboratory personnel and the administration at Livingstone University Teaching Hospital for the support given during the study.

## References

1. Kombe Kombe AJ, Li B, Zahid A, Mengist HM, Bounda GA, Zhou Y, et al. Epidemiology and Burden of Human Papillomavirus and Related Diseases, Molecular Pathogenesis, and Vaccine Evaluation. Frontiers in Public Health [Internet]. 2021 [cited 2022 Sep 29];8. Available from: https://www.frontiersin.org/articles/10.3389/fpubh.2020.552028
2. Dunne EF, Park IU. HPV and HPV-associated diseases. Infect Dis Clin North Am. 2013 Dec;27(4):765–78.
3. Martinelli M, Musumeci R, Sechi I, Sotgiu G, Piana A, Perdoni F, et al. Prevalence of Human Papillomavirus (HPV) and Other Sexually Transmitted Infections (STIs) among Italian Women Referred for a Colposcopy. Int J Environ Res Public Health. 2019 Dec 9;16(24):E5000.
4. Kombe Kombe AJ, Li B, Zahid A, Mengist HM, Bounda GA, Zhou Y, et al. Epidemiology and Burden of Human Papillomavirus and Related Diseases, Molecular Pathogenesis, and Vaccine Evaluation. Frontiers in Public Health [Internet]. 2021 [cited 2022 Sep 22];8. Available from: https://www.frontiersin.org/articles/10.3389/fpubh.2020.552028
5. Seyoum A, Assefa N, Gure T, Seyoum B, Mulu A, Mihret A. Prevalence and Genotype Distribution of High-Risk Human Papillomavirus Infection Among Sub-Saharan African Women:

A Systematic Review and Meta-Analysis. Frontiers in Public Health [Internet]. 2022 [cited 2022 Oct 24];10. Available from: https://www.frontiersin.org/articles/10.3389/fpubh.2022.890880

6. De Vuyst H, Alemany L, Lacey C, Chibwesha CJ, Sahasrabuddhe V, Banura C, et al.

The Burden of Human Papillomavirus Infections and Related Diseases in Sub-Saharan Africa. Vaccine. 2013 Dec 29;31(0 5):F32–46.

7. Bray F, Ferlay J, Soerjomataram I, Siegel RL, Torre LA, Jemal A. Global cancer statistics 2018: GLOBOCAN estimates of incidence and mortality worldwide for 36 cancers in 185 countries. CA: A Cancer Journal for Clinicians. 2018;68(6):394–424.
8. Arbyn M, Weiderpass E, Bruni L, de Sanjosé S, Saraiya M, Ferlay J, et al. Estimates of incidence and mortality of cervical cancer in 2018: a worldwide analysis. Lancet Glob Health. 2019 Dec 4;8(2):e191–203.
9. Mboumba Bouassa RS, Prazuck T, Lethu T, Jenabian MA, Meye JF, Bélec L. Cervical cancer in sub-Saharan Africa: a preventable noncommunicable disease. Expert Rev Anti Infect Ther. 2017 Jun;15(6):613–27.
10. Mboumba Bouassa RS, Prazuck T, Lethu T, Meye JF, Bélec L. Cervical cancer in subSaharan Africa: an emerging and preventable disease associated with oncogenic human papillomavirus. Med Sante Trop. 2017 Feb 1;27(1):16–22.
11. Kalima M, Lishimpi K, Meza JL, Watanabe-Galloway S, Msadabwe SC, Mwaba CK, et al. Observed and Expected Incidence of Cervical Cancer in Lusaka and the Southern and Western Provinces of Zambia, 2007 - 2012. Int J Gynecol Cancer. 2015 Jan;25(1):98–105.
12. Kalubula M, Shen H, Makasa M, Liu L. Epidemiology of Cancers in Zambia: A significant variation in Cancer incidence and prevalence across the nation. Malawi Med J. 2021 Sep;33(3):186–95.
13. Banda L, Nyirongo T, Muntanga M. Cancer – An Emerging Health Problem:The Zambian Perspective. Health Press Zambia Bull. 2019;2-4.
14. WHO. Costing the National Strategic Plan On Prevention and Control of Cervical Cancer: Zambia, 2019 to 2023. World Health Organisation; 2020.
15. Hayumbu V, Hangoma J, Hamooya BM, Malumani M, Masenga SK. Cervical cancer and precancerous cervical lesions detected using visual inspection with acetic acid at Livingstone Teaching Hospital. Pan Afr Med J. 2021;40:235.
16. Rodríguez-Álvarez MI, Gómez-Urquiza JL, Husein-El Ahmed H, Albendín-García L,

Gómez-Salgado J, Cañadas-De la Fuente GA. Prevalence and Risk Factors of Human Papillomavirus in Male Patients: A Systematic Review and Meta-Analysis. Int J Environ Res Public Health. 2018 Oct 10;15(10):2210.

17. Tounkara FK, Téguété I, Guédou FA, Talbot D, Traoré CB, Béhanzin L, et al. Typespecific incidence, persistence and factors associated with human papillomavirus infection among female sex workers in Benin and Mali, West Africa. Int J Infect Dis. 2021 May;106:348– 57.
18. Lekoane KMB, Kuupiel D, Mashamba-Thompson TP, Ginindza TG. The interplay of HIV and human papillomavirus-related cancers in sub-Saharan Africa: scoping review. Syst Rev. 2020 Apr 22;9(1):88.
19. Liu G, Mugo NR, Brown ER, Mgodi NM, Chirenje ZM, Marrazzo JM, et al. Prevalent human papillomavirus infection increases the risk of HIV acquisition in African women:

advancing the argument for human papillomavirus immunization. AIDS. 2022 Feb 1;36(2):257– 65.

20. Shi R, Devarakonda S, Liu L, Taylor H, Mills G. Factors associated with genital human papillomavirus infection among adult females in the United States, NHANES 2007-2010. BMC Res Notes. 2014 Aug 18;7:544.
21. Syrjänen S. Human papillomavirus infection and its association with HIV. Adv Dent Res. 2011 Apr;23(1):84–9.
22. Ramogola-Masire D, Luckett R, Dreyer G. Progress and challenges in human papillomavirus and cervical cancer in southern Africa. Curr Opin Infect Dis. 2022 Feb 1;35(1):49–54.
23. Ng’andwe C, Lowe JJ, Richards PJ, Hause L, Wood C, Angeletti PC. The distribution of sexually-transmitted Human Papillomaviruses in HIV positive and negative patients in Zambia, Africa. BMC Infect Dis. 2007 Jul 16;7(1):77.
24. Sahasrabuddhe VV, Mwanahamuntu MH, Vermund SH, Huh WK, Lyon MD, Stringer JSA, et al. Prevalence and distribution of HPV genotypes among HIV-infected women in Zambia. Br J Cancer. 2007 May 7;96(9):1480–3.
25. Uutela A, Tuomilehto J. Changes in disease patterns and related social trends. Soc Sci Med. 1992 Aug;35(4):389–99.
26. Munn Z, Moola S, Lisy K, Riitano D, Tufanaru C. Methodological guidance for systematic reviews of observational epidemiological studies reporting prevalence and cumulative incidence data. Int J Evid Based Healthc. 2015 Sep;13(3):147–53.
27. LIU G, SHARMA M, TAN N, BARNABAS R. HIV-positive women have higher risk of HPV infection, precancerous lesions, and cervical cancer: A systematic review and metaanalysis. AIDS. 2018 Mar 27;32(6):795–808.
28. Okoye JO, Ofodile CA, Adeleke OK, Obioma O. Prevalence of high-risk HPV genotypes in sub-Saharan Africa according to HIV status: a 20-year systematic review. Epidemiol Health. 2021 May 25;43:e2021039.
29. C B, Jm P. Human papillomavirus in the HIV-infected host: epidemiology and

pathogenesis in the antiretroviral era. Current HIV/AIDS reports [Internet]. 2015 Mar [cited 2023 May 16];12(1). Available from: https://pubmed.ncbi.nlm.nih.gov/25644977/

30. Sc C, M A, G R, Af M, A C, Aa N, et al. HPV and HIV Coinfection in Women from a

Southeast Region of Romania-PICOPIV Study. Medicina (Kaunas, Lithuania) [Internet]. 2022 Jun 3 [cited 2023 May 16];58(6). Available from: https://pubmed.ncbi.nlm.nih.gov/35744023/

31. Vahle K, Gargano JW, Lewis RM, Querec TD, Unger ER, Bednarczyk RA, et al.

Prevalence of Human Papillomavirus Among Women Older than Recommended Age for

Vaccination by Birth Cohort, United States 2003‒2016. J Infect Dis. 2022 Jan 5;225(1):94–104.

32. Clarke MA, Risley C, Stewart MW, Geisinger KR, Hiser LM, Morgan JC, et al. Agespecific prevalence of human papillomavirus and abnormal cytology at baseline in a diverse statewide prospective cohort of individuals undergoing cervical cancer screening in Mississippi. Cancer Med. 2021 Dec;10(23):8641–50.
33. Smith JS, Melendy A, Rana RK, Pimenta JM. Age-specific prevalence of infection with human papillomavirus in females: a global review. J Adolesc Health. 2008 Oct;43(4 Suppl):S525, S25.e1-41.
34. Baussano I, Franceschi S, Gillio-Tos A, Carozzi F, Confortini M, Dalla Palma P, et al. Difference in overall and age-specific prevalence of high-risk human papillomavirus infection in Italy: evidence from NTCC trial. BMC Infect Dis. 2013 May 24;13:238.
35. Pinchoff J, Boyer CB, Mutombo N, Chowdhuri RN, Ngo TD. Why don’t urban youth in Zambia use condoms? The influence of gender and marriage on non-use of male condoms among young adults. PLoS One. 2017 Mar 23;12(3):e0172062.
36. Hlongwa M, Peltzer K, Hlongwana K. Risky sexual behaviours among women of reproductive age in a high HIV burdened township in KwaZulu-Natal, South Africa. BMC Infectious Diseases. 2020 Aug 1;20(1):563.
37. Lam JUH, Rebolj M, Dugué PA, Bonde J, von Euler-Chelpin M, Lynge E. Condom use in prevention of Human Papillomavirus infections and cervical neoplasia: systematic review of longitudinal studies. J Med Screen. 2014 Mar 1;21(1):38–50.
38. Winer RL, Hughes JP, Feng Q, O’Reilly S, Kiviat NB, Holmes KK, et al. Condom Use and the Risk of Genital Human Papillomavirus Infection in Young Women. N Engl J Med. 2006 Jun 22;354(25):2645–54.
39. Grabovac I, Smith L, Yang L, Soysal P, Veronese N, Isik AT, et al. The relationship between chronic diseases and number of sexual partners: an exploratory analysis. BMJ Sex Reprod Health. 2020 Apr 1;46(2):100–7.
40. Itarat Y, Kietpeerakool C, Jampathong N, Chumworathayi B, Kleebkaow P, Aue-Aungkul A, et al. Sexual behavior and infection with cervical human papillomavirus types 16 and 18. Int J Womens Health. 2019;11:489–94.
41. Liu ZC, Liu WD, Liu YH, Ye XH, Chen SD. Multiple Sexual Partners as a Potential Independent Risk Factor for Cervical Cancer: a Meta-analysis of Epidemiological Studies. Asian Pac J Cancer Prev. 2015;16(9):3893–900.
42. Huang Y, Wu X, Lin Y, Li W, Liu J, Song B. Multiple sexual partners and vaginal microecological disorder are associated with HPV infection and cervical carcinoma development. Oncol Lett. 2020 Aug;20(2):1915–21.
43. Charde SH, Warbhe RA. Human Papillomavirus Prevention by Vaccination: A Review Article. Cureus. 14(10):e30037.
44. Burger EA, Kim JJ, Sy S, Castle PE. Age of Acquiring Causal Human Papillomavirus (HPV) Infections: Leveraging Simulation Models to Explore the Natural History of HPV-induced Cervical Cancer. Clin Infect Dis. 2017 Sep 15;65(6):893–9.
45. Smith MA, Hall MT, Saville M, Brotherton JML, Simms KT, Lew JB, et al. Could HPV Testing on Self-collected Samples Be Routinely Used in an Organized Cervical Screening Program? A Modeled Analysis. Cancer Epidemiol Biomarkers Prev. 2021 Feb;30(2):268–77.
46. Saidu R, Kuhn L, Tergas A, Boa R, Moodley J, Svanholm-Barrie C, et al. Performance of Xpert HPV on Self-collected Vaginal Samples for Cervical Cancer Screening Among Women in South Africa. J Low Genit Tract Dis. 2021 Jan 1;25(1):15–21.
47. Katanga JJ, Rasch V, Manongi R, Pembe AB, Mwaiselage JD, Kjaer SK. Concordance in HPV Detection Between Self-Collected and Health Provider-Collected Cervicovaginal Samples Using careHPV in Tanzanian Women. JCO Glob Oncol. 2021 Jun;7:985–91.
48. Asciutto KC, Ernstson A, Forslund O, Borgfeldt C. Self-sampling with HPV mRNA analyses from vagina and urine compared with cervical samples. Journal of Clinical Virology. 2018 Apr 1;101:69–73.
49. Sy F, Greuel M, Winkler V, Bussmann H, Bärnighausen T, Deckert A. Accuracy of HPV testing on self-collected and clinician-collected samples for different screening strategies in African settings: A systematic review and meta-analysis. Gynecologic Oncology. 2022 Aug 1;166(2):358–68.
50. Cho HW, Shim SR, Lee JK, Hong JH. Accuracy of human papillomavirus tests on selfcollected urine versus clinician-collected samples for the detection of cervical precancer: a systematic review and meta-analysis. J Gynecol Oncol. 2021 Oct 7;33(1):e4.
51. Kim SC, Feldman S, Moscicki AB. Risk of human papillomavirus infection in women with rheumatic disease: cervical cancer screening and prevention. Rheumatology (Oxford). 2018 Jul;57(Suppl 5):v26–33.
52. Garland SM, Brotherton JML, Moscicki AB, Kaufmann AM, Stanley M, Bhatla N, et al. HPV vaccination of immunocompromised hosts. Papillomavirus Res. 2017 Jun 3;4:35–8.
53. Thornton C, Somayaji R, Chu A, Parkins MD. Human papillomavirus (HPV) and cervical dysplasia in adult female cystic fibrosis (CF) lung transplant recipients. Thorax. 2022 Jun;77(6):625–7.

Ethics clearance

[Click here to access/download](https://www.editorialmanager.com/pone/download.aspx?id=35536823&guid=c21e5c08-a32e-412c-b5be-03bb5df742a3&scheme=1)

[Supporting Information](https://www.editorialmanager.com/pone/download.aspx?id=35536823&guid=c21e5c08-a32e-412c-b5be-03bb5df742a3&scheme=1)

[SMHS-MU1-2023-37 Lweendo.pdf](https://www.editorialmanager.com/pone/download.aspx?id=35536823&guid=c21e5c08-a32e-412c-b5be-03bb5df742a3&scheme=1)
