# Supplementary material for: Prevalence and correlates of Human Papillomavirus infection in females from Southern Province, Zambia: A cross-sectional study
Source: PLoS One. 2024 Aug 1;19(8):e0299963. doi: 10.1371/journal.pone.0299963 (PMC11293658; doi:10.1371/journal.pone.0299963)
Supplement: Attachment — Submitted filename: Response to Reviewers.docx [file pone.0299963.s004.docx]

Mulungushi University,

P.O Box 60009,

Livingstone.

23^rd^ April 2024.

The Reviewers,

PLOS ONE Journal.

Dear PLOS ONE reviewers,

**RE: RESPONSE TO REVIEWER COMMENTS**

Thank you so much for reviewing our manuscript and providing invaluable reviews. We appreciate your review comments which we believe have improved our manuscript's readability and credibility.

We have taken time to correct our manuscript and provided a manuscript copy with track changes to reflect our work on the manuscript.

Below are our responses to the review comments.

When submitting your revision, we need you to address these additional requirements.

1. Please ensure that your manuscript meets PLOS ONE's style requirements, including those for file naming. The PLOS ONE style templates can be found at 
<https://journals.plos.org/plosone/s/file?id=wjVg/PLOSOne_formatting_sample_main_body.pdf> and 
<https://journals.plos.org/plosone/s/file?id=ba62/PLOSOne_formatting_sample_title_authors_affiliations.pdf>

**Response from authors:** Thank you. We have referred to the comments and have since adjusted the naming of the manuscript and supporting documents to PLOS ONE’s style requirements.

2. We note that your Data Availability Statement is currently as follows: All relevant data are within the manuscript and its Supporting Information files

Please confirm at this time whether or not your submission contains all raw data required to replicate the results of your study. Authors must share the “minimal data set” for their submission. PLOS defines the minimal data set to consist of the data required to replicate all study findings reported in the article, as well as related metadata and methods (<https://journals.plos.org/plosone/s/data-availability#loc-minimal-data-set-definition>).

**Response from authors:** Thank you, we have since attached the raw data to this submission.

3. Your ethics statement should only appear in the Methods section of your manuscript. If your ethics statement is written in any section besides the Methods, please move it to the Methods section and delete it from any other section. Please ensure that your ethics statement is included in your manuscript, as the ethics statement entered into the online submission form will not be published alongside your manuscript.

**Response from authors:** Thank you, we have since deleted the ethics statement in other parts of the manuscript and only retained it in the methods section.

4. Please include captions for your Supporting Information files at the end of your manuscript, and update any in-text citations to match accordingly. Please see our Supporting Information guidelines for more information: <http://journals.plos.org/plosone/s/supporting-information>.

**Response from authors:** Thank you, we have since made the captions as per recommendation.

5. Please review your reference list to ensure that it is complete and correct. If you have cited papers that have been retracted, please include the rationale for doing so in the manuscript text, or remove these references and replace them with relevant current references. Any changes to the reference list should be mentioned in the rebuttal letter that accompanies your revised manuscript. If you need to cite a retracted article, indicate the article’s retracted status in the References list and also include a citation and full reference for the retraction notice.

**Response from authors:** Thank you, we have reviewed our reference list as recommended. We have not cited any retracted article.

5. Review Comments to the Author

Please use the space provided to explain your answers to the questions above. You may also include additional comments for the author, including concerns about dual publication, research ethics, or publication ethics. (Please upload your review as an attachment if it exceeds 20,000 characters)

Reviewer #1: Thank you for asking me to review the article titled “Prevalence and correlates of Human Papillomavirus infection in females from Southern Province, Zambia: A cross-sectional study”.

The study was a cross sectional retrospective study conducted in Zambia. It involved collection of data on 4, 612 included participants that had been tested for HPV infection between September 2021 and August 2022 using the Aptima® HPV kit on the Hologic Panther platform. The aim was to determine the prevalence and correlates of HPV infection among women from Southern province using programmatic data to generate hypotheses for follow-up interventional studies. The primary outcome was 35.56% of the participants had HPV infection. Significantly age, HIV status and method of sample collection were important correlates for HPV infection among the studied population.

I must commend the team on the research work, as the area of study is of clinical significance to women’s health. Their study contributes to the knowledge of the overall prevention of cervical cancer and has been properly conducted.
The study methodology was sound with ease of reproducibility. The data analysis was comprehensive, and the study design was appropriate. I noted minor corrections, but these do not affect the acceptance of the article.
1. Appropriate punctuation marks are required as some have been omitted.

**Response from authors:** Thank you so much. We have since worked on the punctuation marks as recommended.

2. They chose to use the Wilcoxon rank sum text in place of the initially stated Shapiro-Wilk test, this should be clarified.

**Response from authors:** Thank you. We apologize for the omission, we should have indicated that we used the Shapiro-Wilk test to test for normality, and used Wilcoxon rank sum test to test for the relationship between the medians of the two outcome groups. We have since included this statement.

3. The authors should review the entire manuscript for grammar, typo and punctions.

Overall, the study presents results of an original research with a retrospective cross-sectional design on the Prevalence and correlates of Human Papillomavirus infection in females from Southern Province, Zambia. The methods have been documented properly with ease of reproducibility and with sufficient detail. The results have been represented appropriately and the conclusion was written to support results from the data. The article was well written with good scientific flow. The research integrity was sufficient. I recommend the manuscript be accepted. Thank you.

**Response from authors:** Thank you so much. We have since worked on the typos and punctuation marks as recommended. We have attached the manuscript with the tracked changes for your review.

Reviewer #2: The authors should review and provide a detailed description of the results presented in Table 1. The table currently does not include all participants and lacks an explanation for this omission. It would be helpful if the authors could include a guideline or statement/checklist to ensure transparency and provide a comprehensive description of the entire study.

**Response from authors:** Thank you, we have since reviewed Table 1, and further provided a detailed description accounting for the missing variable entries. Note that we have also added a column (n) to account for the missing variable entries in Table 1.

Yours,

Lweendo Muchaili

On behalf of the authors
